# Pregnant individual’s lived experience of cannabis use during the COVID-19 pandemic: a qualitative study

**DOI:** 10.3389/fpsyt.2023.1161137

**Published:** 2023-04-21

**Authors:** Kelly C. Young-Wolff, Tara R. Foti, Andrea Green, Esti Iturralde, Melanie Jackson-Morris, Monique B. Does, Sara R. Adams, Nancy Goler, Amy Conway, Deborah Ansley, Andrea Altschuler

**Affiliations:** ^1^Division of Research, Kaiser Permanente Northern California, Oakland, CA, United States; ^2^Department of Psychiatry and Behavioral Sciences, University of California, San Francisco, San Francisco, CA, United States; ^3^Sacramento Medical Center, Kaiser Permanente Northern California, Sacramento CA, United States; ^4^Regional Offices, Kaiser Permanente Northern California, Oakland CA, United States

**Keywords:** marijuana, cannabis, COVID-19, pandemic, focus group, pregnancy, prenatal

## Abstract

**Introduction:**

Quantitative studies indicate that the COVID-19 pandemic has contributed to increased rates of prenatal cannabis use. However, little is known about how the pandemic has impacted cannabis use from the perspective of pregnant individuals themselves. Our objective was to characterize COVID-19-related changes in cannabis use among pregnant individuals who used cannabis during the pandemic.

**Methods:**

We conducted 18 focus groups (from 11/17/2021 to 12/17/2021) with Black and White pregnant individuals aged 18+ who self-reported prenatal cannabis use during universal screening at entrance to prenatal care (at ~8 weeks gestation) in Kaiser Permanente Northern California. Virtual focus groups were transcribed and analyzed using thematic analysis.

**Results:**

The sample of 53 pregnant individuals (23 Black, 30 White) was 30.3  years old (SD = 5.2) on average, and most (70%) self-reported daily versus weekly or monthly prenatal cannabis use. Major themes regarding the impact of the pandemic on cannabis use included increases in use (resulting from depression, anxiety, stress, boredom), and changes in social use (less sharing of smoked cannabis products), modes of use (from smoking to other modes due to respiratory concerns) and source (from storefront retailers to delivery).

**Conclusion:**

Coping with mental health symptoms and stress were identified drivers of perceived pandemic-related increases in prenatal cannabis use in 2021. Pregnant individuals adapted their use in ways consistent with public health recommendations to decrease social contact and reduce or quit smoking to mitigate COVID-19 transmission and harms. Proactive, mental health outreach for pregnant individuals during future pandemic waves may reduce prenatal cannabis use.

## 1. Introduction

Cannabis is the most commonly used federally illicit substance during pregnancy, and the prevalence and frequency of prenatal cannabis use have increased in recent years (1, 2). Epidemiologic studies have found that prenatal cannabis use is elevated among pregnant individuals with diagnoses of nausea and vomiting, depressive disorders, anxiety disorders, and trauma (3, 4). Existing qualitative studies indicate that pregnant individuals self-report using cannabis as a way to cope with medical and mental health symptoms, including pain, sleep problems, morning sickness, stress and depressed mood (5–7). Rising rates of prenatal cannabis use are a significant public health problem (1, 2, 8, 9). Cannabis use during pregnancy is associated with potential health risks, including low birthweight and potential neurodevelopmental problems for offspring exposed *in utero* (10–14).

The COVID-19 pandemic, which started in the Spring of 2020, has resulted in increased psychological distress, depression, and substance use among US adults (15–19). Pregnant individuals have faced unique pandemic-related challenges, including major changes to prenatal care, difficulty obtaining childcare, and concerns about the impact of COVID-19 on their pregnancy (20–24). Recent research suggests that rates of cannabis use during pregnancy have increased during the COVID-19 pandemic (25), and pregnant individuals may be using cannabis in an attempt to cope with pandemic-related mental health symptoms. However, qualitative studies that highlight how the pandemic has impacted prenatal cannabis use from the perspective of pregnant individuals themselves are lacking.

To address this gap in the literature, we conducted focus groups at the end of 2021 with Black and White pregnant individuals who self-reported cannabis use during early pregnancy in California, where cannabis is fully legal for adults over the age of 21. Results from these focus groups allow us to understand the impact of the pandemic on individuals with lived experience of prenatal cannabis use.

## 2. Materials and methods

The study took place in Kaiser Permanente Northern California (KPNC)’s large multispecialty healthcare system serving >4.5 million diverse members (26), and was approved by the KPNC Institutional Review Board. English-speaking pregnant adults aged ≥18 who self-reported non-Hispanic Black or non-Hispanic White race/ethnicity in the electronic health record and self-reported any cannabis use since pregnancy on the self-administered Prenatal Screening Questionnaire as part of universal screening done at entrance to prenatal care (at ~8 weeks gestation) were eligible; those who used daily or weekly were prioritized for recruitment. For this initial study we selected pregnant individuals who were non-Hispanic Black or non-Hispanic White because they constitute the racial/ethnic groups with the highest prevalence of prenatal cannabis use in our healthcare system (27). We did not utilize electronic health record data on prenatal cannabis use based on routine urine toxicology testing done at entrance to prenatal care because we wanted to recruit participants who were willing to self-disclose prenatal cannabis use and would be more likely to feel comfortable discussing this topic in a focus group setting.

After conducting chart reviews to confirm that the patient had no documented pregnancy loss, patients were sent an email with information about the study and an option to opt out. Potential participants were then contacted by phone and provided verbal informed consent to participate in this study. The KPNC *IRB waived the requirement* to obtain signed consent as the research presented no more than minimal risk of harms to participants and involves no procedures for which written consent is normally required outside of the research context. Patients were informed that participation in the study would be confidential and would not impact their clinical care. During recruitment, patients were asked if they were still using cannabis and if not, the date when they stopped using.

We developed a semi-structured focus group script that included multiple domains, including reasons for prenatal cannabis use, perceived harms, changes in use during pregnancy, and communications with clinicians about prenatal cannabis use. Participants were asked whether they think pregnant women are more likely to use cannabis now than they were 5 years ago and why or why not. Interview probes included the COVID-19 pandemic and individuals could respond about what they have seen among pregnant individuals in general or respond about their own cannabis use behaviors (Supplement). The semi-structured format and allowed for new themes to emerge. HIPAA-compliant virtual focus groups took place *via* video-conferencing software (Microsoft Teams) from 11/17/2021 to 12/17/2021. Participants were encouraged, but not required, to have their cameras on during the focus group. We chose to match focus group leaders and participants on race, with recognition that people with shared experiences may be more open with each other (28, 29), and to acknowledge the role that race/ethnicity plays in the experiences of pregnant individuals. Individuals received a $50 gift card for participating. The study team had weekly meetings to review field notes and to discuss emerging themes. After 18 groups, thematic saturation was achieved. Focus groups were recorded and professionally transcribed. Video and audio were deleted after transcription was completed.

A thematic analysis approach was used to analyze the transcripts. First, three members of the team (KYW, TF, AA) created a codebook after reviewing all transcripts. Next, study team members (KYW, TF, AA, EI, MD) independently coded two transcripts, and the team further refined the code book to reach consensus on themes and subthemes. The remaining 16 focus groups were manually coded by the study team using NVivo Qualitative Analysis Software (Release 1.6.1). Quotes related to the impact of the COVID-19 pandemic were selected for this study and transcripts were compared for potential differences in responses by participant race. Additional details about the focus group methods appear elsewhere (30).

Descriptive statistics (frequencies, proportions, means) were used to summarize patient socio-demographics, frequency of prenatal cannabis use, whether participants had quit using cannabis at the time of recruitment, and trimester participants quit using cannabis among those who had stopped using.

## 3. Results

Of 304 eligible patients, 139 were unable to be reached, 53 refused, 2 were found to be ineligible, 5 had time conflicts, and 1 did not complete the consent process. Of the 104 individuals who were scheduled for a focus group, 51 did not participate (39 did not show up, 10 canceled, and 2 had groups that were canceled by the group leader) and 53 participated in one of 18 focus groups, including 23 Black individuals and 30 White individuals. The average length of the 18 focus groups was 73.4 min (range 42–92 min) and the number of participants in a focus group ranged from one to six.

Descriptive information is provided in Table 1. The sample (*n* = 53) had a mean age of 30.3 (SD = 5.2) years, 17.0% were in their first trimester, 47.2% were in their second trimester, and 35.8% were in their third trimester at the time of recruitment. At entrance to prenatal care, 69.8% self-reported daily cannabis use and 30.2% reported weekly or monthly or less cannabis use since pregnancy. The median (interquartile range) time from the first prenatal visit to the phone screening was 15.1 weeks (7.6–21.7). Most (69.8%) reported that they had quit using cannabis at the time of study recruitment. Of those who quit, 83.8% quit in the first trimester and 16.2% quit during the second or third trimester.

**Table 1:** Characteristics of focus group participants (*N* = 53).

Participant characteristics	*N*	%
Age categories		
21–25	11	20.8
26–30	19	35.8
31–35	13	24.5
36–40	10	18.9
Race		
Black	23	43.4
White	30	56.6
Trimester at phone screening		
1st	9	17.0
2nd	25	47.2
3rd	19	35.8
Frequency of self-reported cannabis use during pregnancy		
Daily	37	69.8
Weekly/Monthly	16	30.2
Trimester the participant stopped using cannabis		
1st	31	58.5
2nd or 3rd	6	11.3
N/A – still using	16	30.2

We identified six themes related to the COVID-19 pandemic in the following two domains: (1) Impact of mental health and isolation/boredom on cannabis use during the pandemic, and (2) Changes in specific cannabis-related behaviors. Themes were consistent across focus groups with Black and White participants, although comparatively fewer Black participants discussed the impact of the pandemic than did White participants.

### 3.1. COVID-19 related changes in prenatal cannabis use

#### 3.1.1. Mental health-related increases in cannabis use

Many participants described how the COVID-19 pandemic has led to greater cannabis use during pregnancy. Increased psychological and financial distress, depression, and anxiety were identified as drivers of COVID-19 related increases in cannabis use. One participant noted, “COVID, the pandemic itself was really stressful, and I feel like even people not pregnant, they using you know cannabis to help with the stress …. I feel like everybody gonna have like some type of PTSD from all of this.” Another highlighted the impacts of financial distress resulting from the pandemic: “So for those people who were not working or the stress of the money, and I know the economy was really a mess for a while …. I think that might have heightened cannabis usage just in people trying to relax, calm down, take a breather, and not have to deal with the intenseness of COVID, especially at the height of it.”

Several participants described how the impacts of the pandemic on cannabis use changed over time. One participant who was pregnant twice during the pandemic described how changes in her pandemic-related anxiety differentially impacted her cannabis use during her first versus second pregnancy, noting: “[During my first pregnancy] my reasoning for smoking was to help me wind down from my workday, and I wasn’t attending work as much, and I was just able to stay home, so I actually did not feel like I needed it. Whereas my second pregnancy now, I am back at work, but we have these masks, and some people aren’t vaccinated, I’m definitely sensing a lot more anxiety this time around …. so it’s been a lot harder to quit cannabis this time around.” Several participants noted that cannabis use was most affected during the early months of the pandemic. One reported, “I think the pandemic does increase use of cannabis during pregnancy. Maybe not this far into COVID but I think especially early on when quarantine was a lot more serious, and people who are social and do recharge their batteries by being social were having to be isolated and depression was an even bigger issue. Or people becoming very anxious and going stir crazy being stuck in the house, and then finding out oh, I’m pregnant, and all these things that I would have wanted to do during pregnancy, I cannot do. So, becoming a coping mechanism.”

#### 3.1.2. Isolation/boredom-related increases in cannabis use

Some participants reported that isolation and boredom resulting from pandemic-related social-distancing measures contributed to increases in cannabis use during the COVID-19 pandemic. One participant described: “I think that, yeah, the pandemic leads more people to using cannabis because it’s something to do and being stuck at home during quarantine definitely sucked. I work from home. I still have those days where I’m like, ‘I just need to go out and do something. I have nothing to do at home that I want to do’.” Another noted: “I’m one of the type of people that it’s hard for me without a routine. So, I’m definitely more likely to smoke more, to drink more if I’m still at home, which I am.”

Participants also highlighted the unique challenges of the pandemic for pregnant individuals who are already advised to monitor or change their health behaviors as part of standard obstetric care. One reported: “[With the pandemic] going on like 2 years now, where you might be [in a routine of] drinking more at home or smoking more at home, and your whole life has changed, it’s harder to get out of, we are already so limited. We’ve been so limited now. And during pregnancy, you are supposed to be even more limited. So, I think that would make it even more difficult [to quit cannabis use during pregnancy], you know, once you are into those new routines.”

#### 3.1.3. Little impact or decreases in cannabis use

Conversely, some participants described how the pandemic had little impact on cannabis use or even helped them to abstain from using cannabis. One woman emphasized how the experience of being pregnant transcends any impacts of the pandemic: “I think prenatal cannabis use would be the same if there wasn’t a pandemic because women are still going through the same issues regardless of a pandemic or not. Building a baby inside of you does not change because the world is falling apart around you. That has nothing to do with it. It might slightly impact the way you are handling things around you so maybe that could add some extra stress and anxiety, but I do not necessarily think that being in a pandemic has changed the troubles of being pregnant.” Another noted that social distancing resulting from the pandemic has made it easier for her to abstain from cannabis use during pregnancy: “I’ve been lucky that you know, with the pandemic, I have not been around friends and family smoking. And I know that it would be a big struggle for me if I was smelling it and watching it. The biggest thing that makes me want to smoke is watching other people smoke, even if they are smoking cigarettes, it makes me want to do the act of smoking.”

Some participants reported that their cannabis use during pregnancy was not impacted by the pandemic because they were not pregnant during the height of the pandemic. For example, one participant stated, “It [the pandemic] did not really change anything for me. Like my pregnancy kind of started after the height of COVID. Yeah. So I do not really feel like it affected it at all.” Another discussed how her cannabis use increased during the first year of the pandemic prior to her pregnancy: “I was sitting at home, and I was bored…. And it was like, ‘Oh, I can do this. Like, I’m here! I could sit here. I could eat all day. I could smoke all day. I’m here for a whole year.’ I definitely was smoking more, like that whole entire year. Definitely. But I wasn’t pregnant.”

### 3.2. COVID-19 related changes in cannabis use behaviors and source of cannabis

#### 3.2.1. Changes in sharing smoked cannabis products

A few participants described how they stopped sharing smoked cannabis products (e.g., pipes, joints, etc.) due to concerns about COVID-19. One noted: “We stopped sharing joints because we did not know. We did not know the risks. We did not know how easily it could spread….So, I mean, if it affected anything, it maybe just made us a little more cautious in sharing things…. If we’d still get together and smoke, we would just use our own things and not share because you never know.” Others described how they returned to pre-pandemic sharing behaviors after their friends were vaccinated. One noted: “I think before we got vaccinated, we were like, ‘Nah, we do not want to share with you.’ Now, all of our friends are vaccinated, like now we do not really care as much.”

#### 3.2.2. Changes in mode of cannabis administration

Several participants perceived smoking to be a risk factor for COVID-19 and reported switching from smoking cannabis to other modes of cannabis administration that they viewed to be safer with regard to COVID-19. One noted: “And I know that people who smoke any kind of anything can be more at risk for COVID-19. So, for me, I switched over to different methods just because I felt safer.” Another described: “You have the people who are overly concerned about their health and do not want to cause any sort of detriment to their lungs if COVID-19 is a respiratory illness that’s going around….If you are concerned about the respiratory effects, then you would just choose to maybe do edibles instead.”

#### 3.2.3. Changes in source of cannabis products

Some described how concerns about the increased risks of COVID-19 during pregnancy led them to change their source of cannabis products. For example, one participant described switching from purchasing cannabis at a retailer to doing home delivery: “I started doing more home delivery services, as opposed to going to a dispensary just because I did not want to be surrounded by people….I had talked to my doctor about the heightened risks of getting sick while pregnant – I went out of my way to avoid being around more people if I could help it.”

## 4. Discussion

This timely focus group study characterizes the impact of the COVID-19 pandemic on cannabis use from the perspective of pregnant individuals who used cannabis during early pregnancy. Participants generally perceived that pregnant individuals are more likely to use cannabis during the pandemic, primarily driven by increases in anxiety, depression, isolation and boredom. Participants identified cannabis use as a coping mechanism and described how pandemic-related increases in prenatal cannabis use corresponded directly with changes in pandemic-related stress. Similar increases in cannabis use as a result of coping with COVID-19-related emotional and psychological distress have been found in qualitative studies of other vulnerable populations, including young adults (31), and our findings complement prior research showing that pregnant individuals report using cannabis to cope with medical and mental health symptoms during pregnancy (5–7).

Prior studies have shown that the pandemic has had a major impact on pregnant individuals, resulting in increases in depression, anxiety, loneliness, COVID-19-specific worries related to the potential health effects of the COVID-19 on their pregnancy, and concerns about changes to prenatal care (e.g., lack of a support person during delivery) (21, 22, 32). Studies examining the impact of the COVID-19 pandemic on substance use during pregnancy have found that depression symptoms and financial difficulties are associated with a higher likelihood of cannabis use and polysubstance use during pregnancy (33). Recent electronic health record data have documented an increase in rates of prenatal cannabis use from before to during the pandemic (25), and findings from this focus group study provide insights into the potential mechanisms underlying pandemic-related increases in cannabis use during pregnancy.

Importantly, for some, the COVID-19 pandemic had little impact on their likelihood of using cannabis, and for others the isolation of the pandemic provided an ideal respite from common risk factors/triggers for cannabis use (e.g., seeing others smoke). Importantly, some patients felt that their cannabis use behaviors during pregnancy were not impacted because they were not pregnant until later in the pandemic. This perception aligns with research indicating that frequency of cannabis use and self-reported mental distress among US adults increased during the early months of the pandemic and then returned to baseline levels (34). While the study took place more than one and a half years into the pandemic (November and December 2021), the WHO designated the COVID-19 Omicron variant as a “variant of concern” on November 26, 2021, due to increased transmissibility (35), and the potential for another surge. Yet, many participants spoke about the pandemic in the past-tense, or described getting pregnancy after the pandemic, suggesting that most felt like the greatest impacts of the pandemic were behind them. Participants tended to report on COVID-19 related changes in patterns of or reasons for prenatal cannabis use that are applicable to other populations (e.g., stress-related increases in use), rather than on pregnancy-specific impacts (e.g., concerns about potential impacts of COVID-19 on the fetus). It is possible that individuals who were pregnant earlier during the pandemic may have had different experiences and potentially more responses specifically relating to the interaction of the pandemic and pregnancy.

Our findings also highlight how pregnant individuals who used cannabis early in pregnancy adapted their cannabis use behaviors to reduce potential harms, by not sharing cannabis smoked products, switching to non-smoked modes of administration, and changing to delivery vs. entering storefront retailers. These changes in cannabis-related behaviors are consistent with other research in non-pregnant populations (36), and support the notion that pregnant individuals are motivated to live healthier lifestyles to improve the health of their developing child.

### 4.1. Limitations

This study has several limitations. Our sample included pregnant non-Hispanic Black and non-Hispanic White individuals in KPNC, and nearly all reported self-reported daily or weekly (versus less frequent) cannabis use during early pregnancy. Future studies with participants of other racial/ethnic groups, uninsured individuals, and those with less frequent cannabis use during pregnancy, and those living in states where cannabis is not legal are needed to better understand pregnant individual’s perspectives of how the pandemic impacted cannabis use. In addition, consistent with studies showing that cannabis use is highest among pregnant individuals during the first trimester, most participants in our sample reported that they had quit using cannabis at the time of study recruitment, and we are unable to determine whether their self-reported use since pregnancy was only prior to pregnancy recognition. Additional studies are needed to understand the extent to which the COVID-19 pandemic impacted whether pregnant individuals quit or continued cannabis use during pregnancy. Finally, individuals who were willing to participate in the focus group study may have unique perspectives that may not generalize to those who were eligible but were unreachable or chose not to participate; however, we note that focus group studies are not meant to be generalizable and are intended to be hypothesis generating.

## 5. Conclusion

The current study adds novel qualitative data suggesting that increased depression, anxiety, isolation and boredom are perceived drivers of pandemic-related increases in prenatal cannabis use. Results highlight the need for strategies and programs that combat these issues to potentially decrease prenatal cannabis use and increase positive coping. Most pregnant individuals have regular contact with a healthcare system, even during the COVID-19 pandemic, and clinicians and healthcare systems can help to support pregnant individuals by providing non-judgmental information about the health effects of prenatal cannabis use, taking time to understand reasons for cannabis use, and linking pregnant patients with resources tailored to their specific needs. Further, early comprehensive, and routine screening for prenatal anxiety and depression during the pandemic, along with linkage to resources and interventions, may hold promise for helping pregnant individuals cope with the significant mental health impacts of the pandemic in ways that do not involve cannabis use. Finally, results underscore the impact of social distancing on pregnant women, and suggest that group-based prenatal care, and public health interventions that offer suggestions and strategies for combatting isolation in future pandemic waves may be particularly beneficial.

## Data availability statement

The original contributions presented in the study are included in the article/supplementary material, further inquiries can be directed to the corresponding author.

## Ethics statement

The studies involving human participants were reviewed and approved by The Kaiser Permanente Northern California Institutional Review Board. Patients provided verbal consent and the ethics committee waived the requirement of written consent for participants.

## Author contributions

TF, AG, EI, and KY-W created the interview guide. TF and AG led the focus groups. AA, TF, and KY-W developed the coding scheme. AA, TF, EI, KY-W, and MD reviewed and coded the transcripts. KY-W drafted the manuscript and obtained funding. All authors provided critical revisions to the manuscripts and approved the submitted version.

## Funding

This study was supported by a NIH NIDA K01 Award (DA043604), a KPNC Health Equity Supplement, and The Permanente Medical Group (TPMG) Delivery Science Fellowship Program.

## Conflict of interest

The authors declare that the research was conducted in the absence of any commercial or financial relationships that could be construed as a potential conflict of interest.

## Publisher’s note

All claims expressed in this article are solely those of the authors and do not necessarily represent those of their affiliated organizations, or those of the publisher, the editors and the reviewers. Any product that may be evaluated in this article, or claim that may be made by its manufacturer, is not guaranteed or endorsed by the publisher.
